# Genetic polymorphism and natural selection of Duffy binding protein of *Plasmodium vivax *Myanmar isolates

**DOI:** 10.1186/1475-2875-11-60

**Published:** 2012-03-01

**Authors:** Hye-Lim Ju, Jung-Mi Kang, Sung-Ung Moon, Jung-Yeon Kim, Hyeong-Woo Lee, Khin Lin, Woon-Mok Sohn, Jin-Soo Lee, Tong-Soo Kim, Byoung-Kuk Na

**Affiliations:** 1 Department of Parasitology and Institute of Health Sciences, Gyeongsang National University School of Medicine, Jinju 660-751, Korea; 2 Department of Anatomy, Yonsei University College of Medicine, Seoul 120-752, Korea; 3 Division of Malaria and Parasitic Diseases, National Institute of Health, Korea Centers for Disease Control and Prevention, Osong 122-701, Korea; 4 Department of Pathology, University of Florida, J-566, 1600 SW Archer Road, Gainesville, FL 32610, USA; 5 Department of Health, Vector Borne Diseases Control Project, 36 Theinbyu Road, Mandalay, Myanmar; 6 Department of Internal Medicine and Inha Research Institute for Medical Sciences, Inha University School of Medicine, Incheon 400-712, Korea; 7 Department of Parasitology and Inha Research Institute for Medical Sciences, Inha University School of Medicine, Incheon 400-712, Korea

**Keywords:** *Plasmodium vivax*, Duffy binding protein, Myanmar

## Abstract

**Background:**

*Plasmodium vivax *Duffy binding protein (PvDBP) plays an essential role in erythrocyte invasion and a potential asexual blood stage vaccine candidate antigen against *P. vivax*. The polymorphic nature of PvDBP, particularly amino terminal cysteine-rich region (PvDBPII), represents a major impediment to the successful design of a protective vaccine against vivax malaria. In this study, the genetic polymorphism and natural selection at PvDBPII among Myanmar *P. vivax *isolates were analysed.

**Methods:**

Fifty-four *P. vivax *infected blood samples collected from patients in Myanmar were used. The region flanking PvDBPII was amplified by PCR, cloned into *Escherichia coli*, and sequenced. The polymorphic characters and natural selection of the region were analysed using the DnaSP and MEGA4 programs.

**Results:**

Thirty-two point mutations (28 non-synonymous and four synonymous mutations) were identified in PvDBPII among the Myanmar *P. vivax *isolates. Sequence analyses revealed that 12 different PvDBPII haplotypes were identified in Myanmar *P. vivax *isolates and that the region has evolved under positive natural selection. High selective pressure preferentially acted on regions identified as B- and T-cell epitopes of PvDBPII. Recombination may also be played a role in the resulting genetic diversity of PvDBPII.

**Conclusions:**

PvDBPII of Myanmar *P. vivax *isolates displays a high level of genetic polymorphism and is under selective pressure. Myanmar *P. vivax *isolates share distinct types of PvDBPII alleles that are different from those of other geographical areas. These results will be useful for understanding the nature of the *P. vivax *population in Myanmar and for development of PvDBPII-based vaccine.

## Background

*Plasmodium vivax *Duffy binding protein (PvDBP) is one of the erythrocyte-binding proteins, which belongs to the large erythrocyte binding protein family [1]. PvDBP is expressed on the merozoite of *P. vivax *and plays an essential role in erythrocyte invasion of the parasite by mediating irreversible binding with its corresponding receptor, the duffy antigen receptor for chemokines (DARC), on the surface of erythrocytes [1-4]. Similar to other plasmodial proteins known to participate in such processes, PvDBP is suggested to be an important vaccine candidate antigen, because it elicits strong immune responses in humans [5,6]. Experimental evidences that antibodies against PvDBP inhibit the interaction of this protein with DARC *in vitro *and block the invasion of *P. vivax *into human erythrocytes also support the notion that this protein is a potential asexual blood stage vaccine candidate antigen against *P. vivax *[7-9].

PvDBP is divided into seven regions (regions I-VII), and the amino terminal cysteine-rich region, region II (PvDBPII), contains the central binding motifs necessary for adherence to DARC [10-12]. Critical binding motifs in PvDBPII have been mapped to a 170 amino acid stretch (amino acids 291-460), which includes cysteines 5-8 [11,12]. PvDBPII shows the highest genetic diversity compared to the remaining PvDBP regions and appears to be under strong selective pressure [13,14]. Analysis of genetic variation of PvDBPII among *P. vivax *field isolates from different geographical regions, including Brazil, Colombia, South Korea, Papua New Guinea, Thailand, showed that the PvDBPII is highly polymorphic, but the cysteine residues are conserved within and between *P. vivax *populations from different geographic regions [14-21]. Although it has been suggested that these polymorphisms do not significantly alter host-parasite binding [17,22], some of them alter immune recognition of PvDBP [23] and most of the PvDBP-specific antibodies detected in infected individuals recognize PvDBPII, rather than other PvDBP regions [7,15]. Consequently, the polymorphic nature of PvDBP, particularly PvDBPII, represents a major impediment to the successful design of a protective vaccine against vivax malaria [14]. Therefore, understanding the nature and genetic polymorphism in PvDBPII among *P. vivax *isolates from distinct geographic areas, particularly where a large proportion of *P. vivax *infections occurs, is important for the rational design of vaccines against vivax malaria.

In this study, the genetic polymorphism and natural selection of PvDBPII among *P. vivax *isolates from Myanmar were analysed. These results suggest that excessive polymorphism of PvDBPII is found in the filed isolates of *P. vivax *in Myanmar.

## Methods

### Blood samples and DNA preparation

The 54 blood samples used in this study were collected from patients who were infected with *Plasmodium vivax *at Wet-Won Station Hospital, Pyin Oo Lwin township, Mandalay Division, Myanmar between 2004 and 2006 [24]. The confirmation of *P. vivax *infection was performed by microscopic examination of thin and thick blood smears and polymerase chain reaction [24]. Informed consent was obtained from all individuals participating in this study before blood sampling under the protocol approved by the Department of Health, The Union of Myanmar, and the Ethics Committee of the Centers for Disease Control and Prevention, Korea. Genomic DNA was extracted from 200 μl of whole blood using a QIAamp Blood kit (Qiagen, Valencia, CA, USA), according to the manufacturer's instruction.

### Amplification and sequencing analysis of PvDBPII

Amplification of the PvDBPII region was performed by the polymerase chain reaction (PCR) using the specific primers, PvDBPII F: 5'-ACCACGATCTCTAGTGCTATTATA-3' and PvDBPII R: 5'-ATTTGTCACAACTTCCTGAGTATT-3'. The amplification reaction was performed using the following thermal cycling profile: 94°C for 5 min, 30 cycles at 94°C for 1 min, 50°C for 1 min, and 72°C for 1 min, followed by a 72°C extension for 10 min. Ex Taq DNA polymerase (Takara, Otsu, Japan) was used in all PCR reactions to prevent any possible nucleotide mis-incorporation. The PCR product was analysed on a 1.2% agarose gel, gel-purified, and ligated into the T&A cloning vector (Real Biotech Cooperation, Banqiaa City, Taiwan). Each ligation mixture was transformed into *Escherichia coli *DH5α competent cells, and positive clones were screened for the presence of the plasmid with the appropriate insert. The sequencing reaction was performed using the BigDye Terminator Cycle Sequencing Ready Reaction kit in an ABI 377 automatic DNA sequencer (Applied Biosystems, Foster City, CA, USA). To verify the sequences, sequence analysis was performed by analysing at least two clones from each isolate.

### Sequence and phylogenetic analyses

Nucleotide and deduced amino acid sequences were analysed using the EditSeq program and Clustal in the Megalign program, a multiple alignment program of the DNASTAR package (DNASTAR, Madison, WI, USA). The phylogeny tree was constructed using the neighbourjoining method with MEGA4 version 4.0 [25]. Bootstrap proportions were used to assess the robustness of the tree with 1,000 bootstrap replications. The sequences reported here have been deposited in the GenBank database under the accession numbers JN255576-JN255587.

### DNA sequence polymorphism analysis

DNA sequence polymorphism analysis was performed on 54 Myanmar PvDBPII sequences. The number of segregating sites (S), haplotype diversity (Hd), nucleotide diversity (π), and average number of pairwise nucleotide differences within the population (*K*) were calculated using the DnaSP ver. 5.10.00 package [26]. The π was also calculated on a sliding window of 100 bases, with a step size of 25 bp to estimate the stepwise diversity across PvDBPII. The rates of synonymous (*K*s) and non-synonymous (*K*n) substitutions were estimated and compared by the Z-test (P < 0.05) in MEGA4 program [25] using the Nei and Gojobori's method [27] with the Jukes and Cantor correction. Tajima's D test [28] and Fu and Li's D and F tests using *P. knowlesi *PvDBPII as an outgroup [29] were performed on DnaSP 5.10.00 to test the neutral theory of evolution.

### Analysis of polymorphism associated with B- and T- cell epitopes

To assess the possibility that diversity in PvDBPII within the Myanmar *P. vivax *isolates may have arisen from host's immune pressure, the genetic diversity in predicted or known B- and T-cell epitopes [14] and MHC binding regions [30] in PvDBPII was examined. Polymorphism of each region was analysed by DnaSP ver. 5.10.00 [26], as described above.

### Recombination parameters and linkage disequilibrium

The recombination parameter (R), which included the effective population size and probability of recombination between adjacent nucleotides per generation, and the minimum number of recombination events (Rm) were measured using DnaSP ver. 5.10.00 [26]. Linkage disequilibrium (LD) between different polymorphic sites was computed in terms of the R^2 ^index.

## Results

### Genetic polymorphisms and amino acid changes

**Figure 1:**
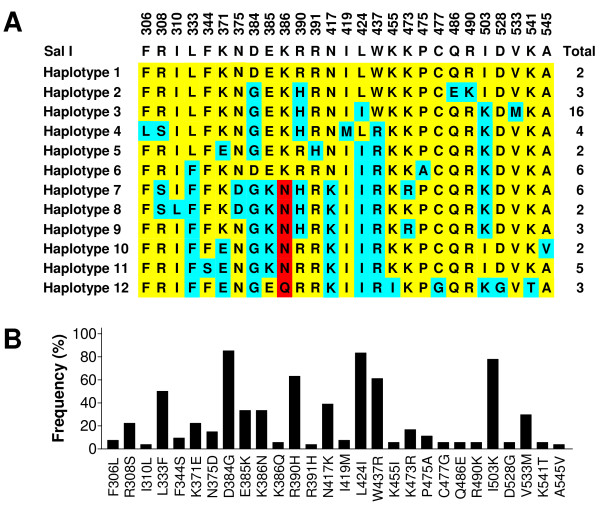
**Sequence polymorphism of PvDBPII in *P. vivax *Myanmar isolates**. (A) Amino acid changes. Polymorphic amino acids are listed for each haplotype. Amino acids identical to those of the reference sequence, Sal I (DQ156512), are marked in yellow. The dimorphic and trimorphic amino acid changes are marked in blue and red, respectively. Total number of sequences for each haplotype is listed in right panel. (B) Frequencies of amino acid changes found in PvDBPII among Myanmar isolates.

**Figure 2:**
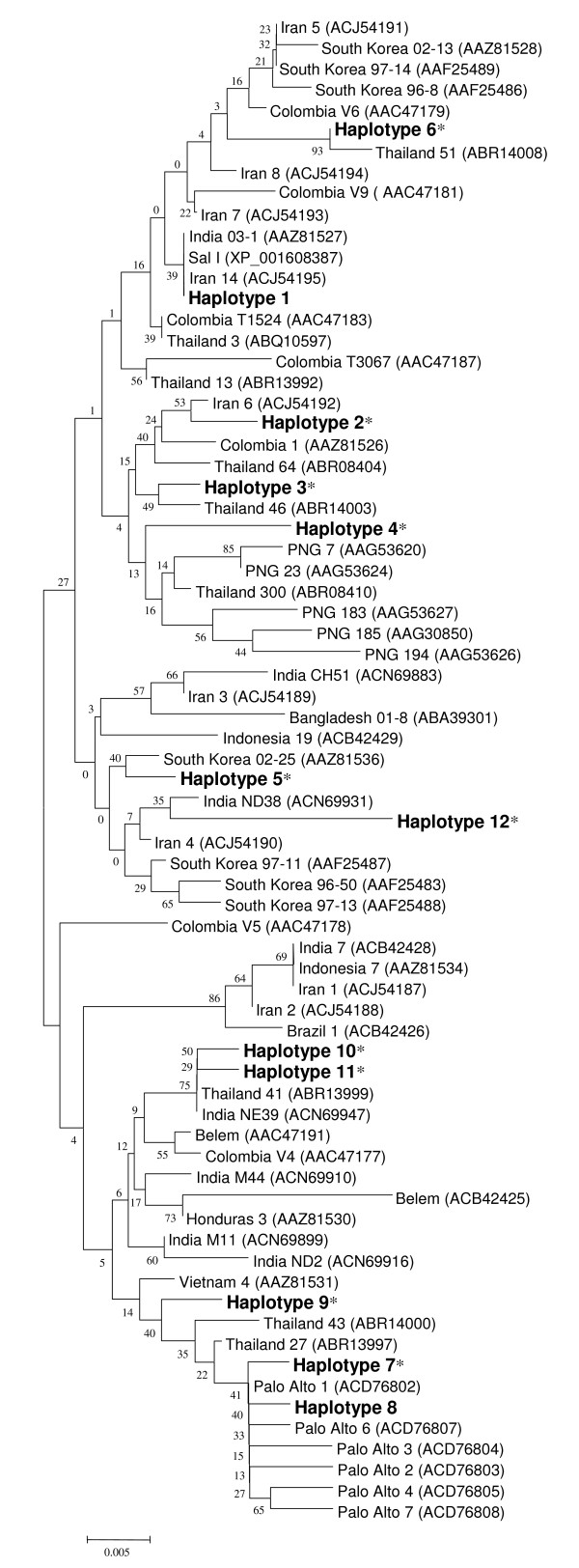
**Phylogenetic analysis**. The phylogenetic tree for the 12 haplotypes of PvDBPII was constructed with a neighbor-joining method using the MEGA4 program. Numbers on the branches indicate bootstrap proportions (1,000 replicates). Numbers on the branches indicate bootstrap proportions (1,000 replicates). The new haplotypes are marked with asterisks.

The region corresponding to PvDBPII was amplified from the 54 Myanmar *P. vivax *isolates by PCR. Each amplified product was cloned into the T&A cloning vector and sequenced in both directions. To verify the sequences, sequencing reactions were performed for at least two plasmid clones for each gene. No size polymorphism was found between the sequences. Analysis and comparison of the sequences against the Sal I (DQ156512) as a reference sequence at the nucleotide level showed that point mutations occurred at 32 positions among the Myanmar isolates. Twelve of these 32 mutations occurred at the first base of the codon, 10 at the second base and 10 at the third base of the codon, resulting in significant amino acid changes (28 non-synonymous and four synonymous mutations) across the PvDBPII among Myanmar isolates. Most of the non-synonymous polymorphisms were non-conservative, resulting in changes in the physico-chemical family of the respective amino acid. A sequence analysis of the deduced amino acid sequences classified them into 12 different haplotypes (haplotypes 1-12) with amino acid changes at 28 positions, in which 1 showed a trimorphic polymorphism (position 386) and the others were dimorphic (Figure 1A). Seventeen of the 28 changes were previously reported, whereas the remaining 11 changes (I310L, F344S, R391H, K455I, K473R, C477G, R490K, D528G, V533M, K541T, and A545V) were new changes that have not been reported previously. Haplotype 3 was predominant (*n *= 16, 29.6%) among the Myanmar isolates. High frequencies of variant amino acids (> 50%), compared to the Sal I sequence, were found for L333F (27/54, 50.0%), D384G (46/54, 85.2%), R390H (34/54, 63.0%), L424I (45/54, 83.3%), W437R (33/54, 61.1%), and I503K (42/54, 77.7%) (Figure 1B). A BLAST search was conducted in the GenBank database to compare these haplotypes with the previously identified PvDBPII sequences. Comparison of the 12 haplotypes found in the Myanmar *P. vivax *isolates with the GenBank sequences revealed that 10 of them (except haplotypes 1 and 8) were novel. Phylogenetic analysis revealed that the 12 Myanmar haplotypes were widely distributed among different isolates from distinct geographic regions (Figure 2). Comparison of the most common variants in PvDBPII among presently studied *P. vivax *populations revealed that Myanmar isolates showed a pattern similar to Thailand isolates [20] but one that differed from Papua New Guinean, Colombian, Brazilian, Iranian, and Sri Lankan isolates [19,21,31] (Table 1). Although the Myanmar isolates showed similar amino acid changes compared to Thailand isolates, nine variants found in the Thailand isolates (R268S, S351C, I367T, S398T, T404R, Q433K, R436T, N507H, and T513K) were not identified in the Myanmar isolates. Meanwhile, 11 variations (I310L, F343S, R391H, K455I, K473R, C477G, R490K, D528G, V533M, K541T, and A545V) found in the Myanmar isolates did not occur in the Thailand isolates.

**Table 1:** Frequencies of the most common variant amino acids in PvDBPII

	F306L	R308S	D384G	K386N	K386Q	N417K	L424I	W437R	S447K	I503K
Myanmar	7.4	22.2	85.2	33.3	5.6	38.9	83.3	61.1	0	77.8
Thailand^a^	6.7	26.7	76.7	40.0	3.0	36.6	86.7	63.3	0	56.7
Iran^b^	5.3	6.6	61.3	6.6	0	44.0	50.6	45.3	0	70.6
Sri Lanka^c^	7.0	13.0	94.0	20.0	0	36.0	49.0	37.0	0	55.0
Papua New Guinea^d^	0	67.0	66.0	8.0	11.0	23.0	34.0	26.0	59.0	29.0
Colombia^d^	0	0	59.0	23.0	0	47.0	47.0	18.0	0	12.0
Brazil^d^	0	12.5	85.0	12.5	0	27.5	32.5	27.5	0	55.0

^a ^[20]; ^b ^[21]; ^c ^[31]; ^d ^[19]

The first letter represents the amino acid in that position in Sal I sequence, and the other letter represents the substituted amino acid

### Nucleotide diversity and natural selection of PvDBPII

**Figure 3:**
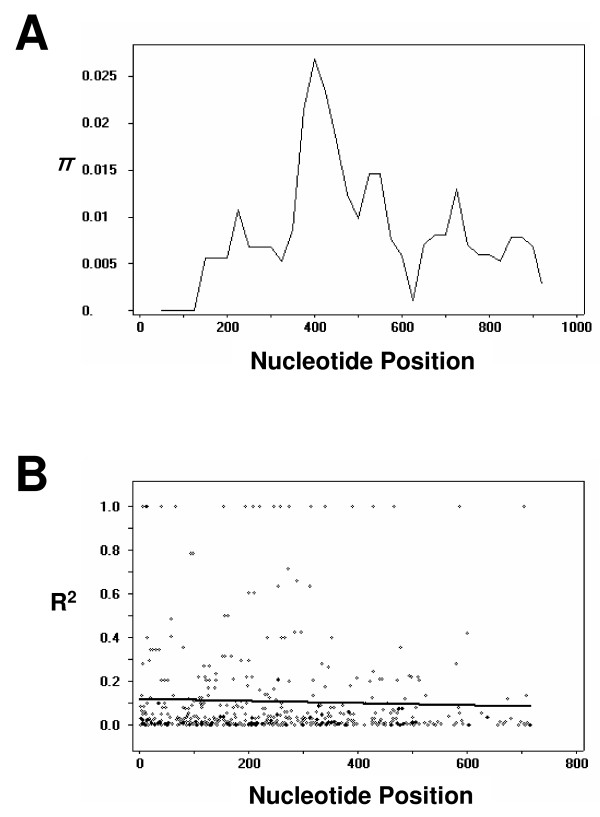
**Natural selection of PvDBPII**. (A) Sliding window plot of nucleotide diversity per site (π) comparing the level of genetic diversity at PvDBPII. The π values were calculated on DnaSP with window length 100 bp and step size of 25 bp. (B) The linkage disequilibrium (LD) plot showing non-random association between nucleotide variants in 54 Myanmar *P. vivax *isolates at different polymorphic sites. The R^2 ^values are plotted against the nucleotide distances with two-tailed Fisher's exact test of significance.

DNA sequence analyses were conducted to determine nucleotide diversity and genetic differentiation at PvDBPII among the Myanmar *P. vivax *isolates. The average number of pairwise nucleotide differences (*K*) for the 963 bp of the PvDBPII region was 7.851 (Table 2). The overall haplotype diversity (Hd) and nucleotide diversity (π) for all 54 sequences were 0.875 ± 0.029 and 0.0079 ± 0.0004, respectively (Table 2). Further analysis, with a sliding window plot (window length 100 bp, step size 25 bp) using the DnaSP package, revealed that π diversity ranged from 0 to 0.027. The two highest peaks of nucleotide diversity within the PvDBPII region were identified between nucleotide positions 370-580 (Figure 3A). To determine whether natural selection contributed to generation of this diversity in PvDBPII within the Myanmar *P. vivax *population, the rate of non-synonymous (*K*n) to synonymous substitutions (*K*s) was estimated using the Nei and Gojobori's method [27], as implemented in the MEGA4 program [25]. The standard error was determined by 1,000 bootstrap replications. The rate of non-synonymous substitutions (*K*n) (0.00917) exceeded the rate of synonymous substitutions (*K*s) (0.00278). The *K*n/*K*s ratio was 3.299 (Table 2). These results suggest that positive natural selection may be occurring in PvDBPII and favoring fixed amino acid replacement in certain areas of the protein. The Tajima's D statistics was 0.264 (P > 0.1), indicating a decrease in population size and/or balancing selection. The Fu and Li's D and F values were 1.867 (P < 0.02) and 1.535 (0.1 > P > 0.05), respectively.

**Table 2:** Estimates of DNA sequence polymorphism and tests of neutrality at PvDBPII among Myanmar isolates

**Total no**. of isolates	Segregating sites (S)	Singleton variable sites	Parsimony informative sites	**Total no**. of mutations	*K*	H	Hd ± SD	π ± SD	*K*n	*K*s	Tajima's
54	32	0	32	32	7.581	12	0.875 ± 0.029	0.0079 ± 0.0004	0.00917	0.00278	0.264 (P > 0.1)

*K*, average number of pairwise nucleotide differences; H, number of haplotypes; Hd, haplotype diversity; π, observed average pairwise nucleotid diversity; *K*n, rate of non-synonymous mutations; *K*s, rate of synonymous mutations

### Polymorphisms associated with B- and T-cell epitopes

To evaluate the association between positive natural selection and host immune evasion, we examined the genetic diversity in predicted or known B- and T-cell epitopes [14] and MHC binding regions [30] in PvDBPII. Most predicted B- and T-cell epitopes and MHC binding regions were polymorphic, and most had a higher rate of non-synonymous mutations (*K*n) than that of synonymous mutations (*K*s) with the exceptions being the regions within peptides 13 and Ic (Table 3). High levels of nucleotide diversity were particularly found in peptides 48 and Ia, which contained spatially proximate polymorphic residues at positions 417, 419, and 427 and positions 385, 386, 390, and 391, respectively, and they showed a clear positive natural selection signature with positive Tajima's D values. In contrast, three peptides (28, 40 and 54), which did not contain known epitopes [14], had lower nucleotide diversity. These results are consistent with the hypothesis that natural selection acts on epitopes in PvDBPII and is related to diversity of PvDBPII [14,30].

**Table 3:** Polymorphism observed in the each epitope sequence

Epitope name	Epitop^a^	Segregating sites (S)	Singleto n variable sites	Parsimony informative sites	**Total no**. of mutations	*K*	H	Hd ± SD	π ± SD	*K*n	*K*s	Tajima's D
5	T/B	3	0	3	3	0.565	4	0.383 ± 0.079	0.013 ± 0.003	0.016	0	-0.289 (P > 0.1)
13	T	0	0	0	0	0	1	0	0	0	0	0
16	T/B	1	0	1	1	0.509	2	0.509 ± 0.013	0.011 ± 0.000	0.015	0	1.741 (P > 0.05)
18	B	1	0	1	1	0.509	2	0.509 ± 0.013	0.011 ± 0.000	0.015	0	1.741 (P > 0.05)
20	T/B	1	0	1	1	0.509	2	0.509 ± 0.013	0.011 ± 0.000	0.014	0	1.741 (P > 0.05)
28	None	0	0	0	0	0	1	0	0	0	0	0
40	None	3	0	3	3	0.867	3	0.542 ± 0.059	0.019 ± 0.003	0.018	0.025	0.642 (P > 0.1)
48	B	5	0	5	5	1.560	6	0.748 ± 0.040	0.035 ± 0.003	0.041	0	1.007 (P > 0.1)
54	None	0	0	0	0	0	1	0	0	0	0	0
66	T	1	0	1	1	0.283	2	0.283 ± 0.068	0.006 ± 0.002	0.008	0	0.382 (P > 0.1)
78	B	1	0	1	1	0.107	2	0.107 ± 0.055	0.002 ± 0.001	0.003	0	-0.675 (P > 0.1)
Ia	MHCIa	3	0	3	3	0.907	4	0.649 ± 0.037	0.034 ± 0.004	0.040	0	0.767 (P > 0.1)
Ib	MHCIb	2	0	2	2	0.214	2	0.107 ± 0.055	0.008 ± 0.004	0.010	0	-0.898 (P > 0.1)
Ic	MHCIc	2	0	2	2	0.492	3	0.458 ± 0.066	0.012 ± 0.002	0.011	0.017	0.212 (P > 0.1)
IIa	MHCIIa	2	0	2	2	0.557	3	0.511 ± 0.043	0.019 ± 0.002	0.021	0.011	0.471 (P > 0.1)
IIb	MHCIIb	2	0	2	2	0.423	3	0.297 ± 0.076	0.016 ± 0.004	0.019	0	-0.064 (P > 0.1)

*K*, average number of pairwise nucleotide differences; H, number of haplotypes; Hd, haplotype diversity; π, observed average pairwise nucleotide diversity; *K*n, rate of non-synonymous mutations; *K*s, rate of synonymous mutations. ^a^B, B-cell epitope; T, T-cell epitope; none, no epitope identified

### Recombination

The minimum number of recombination events between adjacent polymorphic sites (Rm) was 7, whereas R between adjacent sites (Ra) and per gene (Rb) was 0.0314 and 30.2, respectively. The high value of the recombination parameters indicated that high meiotic recombination may occur between sites, resulting in genetic diversity in the PvDBPII. The LD index R^2 ^for the Myanmar population also declined across the analysed region, suggesting that intragenic recombination may also be contributing the increased diversity at PvDBPII (Figure 3B).

## Discussion

Malaria is endemic or hypoendemic in Myanmar and is characterized by the occurrence of all four human-infecting *Plasmodium *species [32]. Although morbidity and mortality rates due to malaria have been declining gradually in recent years, Myanmar still contributes to approximately 60% of malaria deaths in the Southeast Asia [32]. Genetic polymorphisms in the circumsporozoite protein, merozoite surface protein-1 (MSP-1), MSP-3α, and apical membrane antigen-1 (AMA-1) of *P. vivax *Myanmar isolates were analysed previously [24,33,34]. As expected, they showed high levels of genetic polymorphisms, but information on the nature and extent of population diversity within malaria parasites in Myanmar is still limited. In this study, genetic polymorphism and natural selection of PvDBPII in Myanmar *P. vivax *isolates were analysed to expand our knowledge on population diversity of the parasite in the country.

A total of 54 PvDBPII sequences were obtained from Myanmar *P. vivax *isolates. The sequence analysis revealed that the 54 sequences were classified into 12 different haplotypes. A GenBank search for each haplotype revealed that two haplotypes were identical to at least one previously reported PvDBPII sequence found in other regions of the world, whereas the other 10 haplotypes were novel and have not been reported so far. The sequence analysis revealed that a total of 32 point mutations was identified, which resulted in significant amino acid changes (28 non-synonymous and four synonymous changes) through the PvDBPII in Myanmar isolates. Seventeen of the 28 non-synonymous changes were previously reported, whereas the other 11 changes (I310L, F344S, R391H, K455I, K473R, C477G, R490K, D528G, V533M, K541T, and A545V) were unique to Myanmar isolates, which did not hitherto identified. The two highest peaks of nucleotide diversity within PvDBPII in the Myanmar *P. vivax *isolates were identified between C5 and C7, which was consistent with previous observations [14,15,17]. Interestingly, a unique amino acid change (C477G caused by TGT to GGT) was identified in three Myanmar isolates. It is well known that the cysteine residues within PvDBPII are well conserved within and between *P. vivax *populations from different geographic regions studies [14-21,31]. It is not certain the nature of this amino acid change. It can be assumed that this mutation may resulted from a PCR or sequencing error, but to eliminate any possible errors, high quality Taq polymerase with a proof-reading function was used in all amplification processes and sequencing reactions were performed on at least two individual clones for each gene in both directions. But, considering the small number of isolates used in this study, further analyses with a large number of isolates is necessary to confirm this change in the Myanmar *P. vivax *isolates. Taken together, the results of this study suggest that PvDBPII in the Myanmar isolates showed a high level of genetic polymorphism with a nucleotide diversity (π) of 0.0079 ± 0.0004.

Some polymorphic residues in PvDBPII occurred in only one population or geographic region, but common variant amino acids (K371E, D384G, E385K, K386N, N417K, L424I, W437R and I503K) are found in global isolates [19-21,31]. A high frequency (> 50%) of D384G (85.2%), R390H (63.0%), L424I (83.3%), W437R (61.1%), and I503K (77.7%) residues were found in Myanmar isolates compared to that in the Sal I sequence. This was highly similar to a report on Thailand isolates (D384G, 76.7%; R390H, 56.7%; L424I, 86.7%; W437R, 63.3%; I503K, 56.7%) [20] but differed from previous studies showing R308S (67%), D384G (66%), and S447K (59%) in Papua New Guinean isolates; D384G (59%) in Colombian isolates; D384G (85%) and I503K (55%) in Brazilian isolates; D384G (61.3%) and I503K (70.6%) in Iranian isolates; and D384G (94%) and I503K (55%) in Sri Lankan isolates [19,21,31]. F306L, which has only been reported from Asian malaria endemic areas, including Thailand [20], Iran [21], and Sri Lanka [31], was also identified in the Myanmar isolates. Although the Myanmar isolates showed similar amino acid changes compared to those in the Thailand isolates, 9 variants found in the Thailand isolates (R268S, S351C, I367T, S398T, T404R, Q433K, R436T, N507H, and T513K) were not identified in the Myanmar isolates. Meanwhile, 11 variations (I310L, F344S, R391H, K455I, K473R, C477G, R490K, D528G, V533M, K541T, and A545V) found in the Myanmar isolates did not occur in the Thailand isolates. These results indicate that the Myanmar isolates are different from the Thailand isolates even though the two countries are very close geographically.

Although polymorphic residues were widely distributed throughout the PvDBPII sequence, polymorphisms at residues 417, 437, and 503, either in single or in combination, can affect the efficacy of inhibitory antibodies against erythrocyte binding [23,35]. As these residues compose an important discontinuous epitope in PvDBP, which might be the main target for inhibitory antibodies, these polymorphisms could be subject to immune pressure responsible for parasite escape from the host immune system. It has been confirmed that this strong positive selection pressure in PvDBPII promotes greater diversity [14,30]. The immune pressure drives the generation of new PvDBP variants that are still able to bind erythrocytes but become resistant to inhibitory antibodies, suggesting that this DBP region is under positive pressure at critical residues and under negative pressure at the residues involved in receptor recognition [22,23,35]. A low prevalence of variant N417K (38.9%) was observed among Myanmar isolates, but more than 50% of W437R (61.1%) and I503K (77.8%) were identified. Analyses of the combination of variants revealed that W437R-I503K occurred at a higher frequency (70.4%), whereas N417K-W437K and N417K-I503K occurred at frequencies of 38.9% and 25.9%, respectively. This result suggests that there is a strong association between W437R-I503K in PvDBPII in Myanmar *P. vivax *isolates, but not between N417K with either W437R or I503K.

The rate of non-synonymous mutations (*K*n) and that of synonymous mutations (*K*s) is generally used as an indicator of the action of natural selection in most coding gene sequences [27]. Negative selection acting on coding genes can usually be identified when non-synonymous mutations are not advantageous, so the rate of synonymous mutations surpasses that of non-synonymous mutations (*K*s >*K*n). Meanwhile, positive selection is acting on a gene, when non-synonymous mutations can be beneficial (e.g. to avoid the host immune response), and the rate of non-synonymous mutations exceed that of synonymous mutations (*K*n *> K*s). Previous studies on PvDBPII diversity indicate that the high rate of non-synonymous mutations (*K*n) relative to that of synonymous mutations (*K*s) reflects positive selection pressure [14,31,36]. The positive value of *K*n/*K*s (3.299) for all 54 sequences suggest that PvDBPII in Myanmar *P. vivax *isolates is under positive natural selection. The positive values of Tajima's D (0.2635, P > 0.10) and Fu and Li's D (1.867, P < 0.02) and F statistics (1.535, 0.1 > P > 0.05) indicate that PvDBPII alleles occur at more intermediate frequencies than expected and that few alleles are rare or near fixation, which is consistent with the action of balancing selection, which maintains allelic variation in a population. These results collectively suggested that strong balancing selection, probably by host immune selection pressure, occurs at PvDBPII in the Myanmar isolates.

Polymorphism in B- and T-cell epitopes of parasite antigens may well enable parasites to escape host immune responses, as a polymorphism in the epitopes can up or down regulate T-cell responses to the index peptide or completely arrest an immune response, assisting escape of the parasite from the host immune system [37]. The high degree of nucleotide diversity and high rates of non-synonymous to synonymous mutations are observed in known or predicted B- and T-cell epitopes and MHC binding regions of PvDBPII in the Myanmar isolates. Overall nucleotide diversity values for these epitopes and regions were greater than those for the entire PvDBPII. In particular, high levels of nucleotide diversity were identified in peptides 48 and Ia, which is comparable to Brazilian isolates [30]. Positive Tajima's D values for these epitopes also suggested that positive natural selection preferentially acted on the epitopes in PvDBPII in the Myanmar isolates. These epitopes are predicted to be exposed to the surface of the PvDBP molecule [30]. The putative changes in protein structure may alter antibody binding efficacy of a particular epitope, thereby allowing escape from the host protective immune response [13,30].

Many factors may contribute to genetic diversity in malaria populations, including mutations, intragenic recombination, natural selection, gene flow between different regions, and population size. Although it remains controversial, it has been suggested that recombination also contributes to the diversity of PvDBPII [30,36]. The existence of recombination events and the decline in the LD with increasing distance between nucleotide sites suggest that in addition to natural selection meiotic recombination may also contribute to maintain the diversity of PvDBPII among Myanmar *P. vivax *isolates, as reported previously in Brazil, Colombia, and Sri Lanka [30,31].

## Conclusion

The present study is the first report on the genetic polymorphism and natural selection of PvDBPII in Myanmar *P. vivax *isolates. Myanmar *P. vivax *isolates are genetically diverse and this seems to have arisen by a immune evasion mechanism, in which positive natural selection and recombination events maintained the diversity in the form of balancing selection. However, further studies using a larger number of isolates collected from different geographical regions in Myanmar will be helpful to reveal the nationwide parasite heterogeneity and the implementation of malarial control programmes in Myanmar.

## Competing interests

The authors declare that they have no competing interests.

## Authors' contributions

HLJ and JMK performed all the experiments and analysed the sequence data. SUM, JYK, HWL, KL, and BKN collected the blood samples. SUM performed sequence and phylogenetic analyses. BKN and TSK designed the study and supervised the study process. BKN wrote the paper. TSK, WMS, and JSL assisted in writing and editing the manuscript. All authors read and approved the final manuscript.
